# Site-Specific Variation in Radiomic Features of Head and Neck Squamous Cell Carcinoma and Its Impact on Machine Learning Models

**DOI:** 10.3390/cancers13153723

**Published:** 2021-07-24

**Authors:** Xiaoyang Liu, Farhad Maleki, Nikesh Muthukrishnan, Katie Ovens, Shao Hui Huang, Almudena Pérez-Lara, Griselda Romero-Sanchez, Sahir Rai Bhatnagar, Avishek Chatterjee, Marc Philippe Pusztaszeri, Alan Spatz, Gerald Batist, Seyedmehdi Payabvash, Stefan P. Haider, Amit Mahajan, Caroline Reinhold, Behzad Forghani, Brian O’Sullivan, Eugene Yu, Reza Forghani

**Affiliations:** 1 Princess Margaret Hospital, University of Toronto, University Health Network, Toronto, ON M5G 2C1, Canada; xiaoyangxy.liu@mail.utoronto.ca (X.L.); shaohui.huang@rmp.uhn.ca (S.H.H.); brian.osullivan@rmp.uhn.ca (B.O.); 2 Department of Radiology, Brigham and Women’s Hospital, Harvard University, Cambridge, MA 02115, USA; 3 Department of Medical Imaging, University of Toronto, Toronto, ON M5S 1A1, Canada; 4 Augmented Intelligence & Precision Health Laboratory (AIPHL), Department of Radiology and the Research Institute of the McGill University Health Centre, McGill University, Montreal, QC H4A 3J1, Canada; farhad.maleki@mail.mcgill.ca (F.M.); nikesh.muthukrishnan@mail.mcgill.ca (N.M.); katie.ovens@mail.mcgill.ca (K.O.); sahir.bhatnagar@mcgill.ca (S.R.B.); caroline.reinhold@mcgill.ca (C.R.); behzadforghani@outlook.com (B.F.); 5 Princess Margaret Cancer Centre, Department of Radiation Oncology, University of Toronto, Toronto, ON M5S 1A1, Canada; 6 Segal Cancer Centre & Lady Davis Institute for Medical Research, Jewish General Hospital, McGill University, Montreal, QC H3T 1E2, Canada; almudenaperezlara@gmail.com (A.P.-L.); grirs@hotmail.com (G.R.-S.); gerald.batist@mcgill.ca (G.B.); 7 Department of Epidemiology, Biostatistics and Occupational Health, McGill University, Montreal, QC H3A 1A2, Canada; 8 Medical Physics Unit, McGill University, Montreal, QC H3A 1A2, Canada; a.chatterjee@maastrichtuniversity.nl; 9 Division of Pathology, Jewish General Hospital, Montreal, QC H3Y 1E2, Canada; marc.pusztaszeri@mcgill.ca (M.P.P.); alan.spatz@mcgill.ca (A.S.); 10 Section of Neuroradiology, Department of Radiology and Biomedical Imaging, Yale School of Medicine, New Haven, CT 06520, USA; sam.payabvash@yale.edu (S.P.); stefan.haider@yale.edu (S.P.H.); amit.mahajan@yale.edu (A.M.)

**Keywords:** head and neck squamous cell carcinomas, radiomics, machine learning, classification, metastasis, human papilloma virus

## Abstract

**Simple Summary:**

Head and neck squamous cell carcinoma (HNSCC) is the most common mucosal malignancy of the head and neck and a leading cause of cancer death. HNSCC arises from different primary anatomical locations that are typically combined during radiomic analyses assuming that the radiomic features, i.e., quantitative image-based features, are similar based on histopathologic characteristics. However, whether these quantitative features are comparable across tumor sites remains unknown. The aim of our retrospective study was to assess if systematic differences exist between radiomic features based on different tumor sites in HNSCC and how they might affect machine learning model performance in endpoint prediction. Using a population of 605 HNSCC patients, we observed significant differences in radiomic features of tumors from different locations and showed that these differences can impact machine learning model performance. This suggests that tumor site should be considered when developing and evaluating radiomics-based models.

**Abstract:**

Current radiomic studies of head and neck squamous cell carcinomas (HNSCC) are typically based on datasets combining tumors from different locations, assuming that the radiomic features are similar based on histopathologic characteristics. However, molecular pathogenesis and treatment in HNSCC substantially vary across different tumor sites. It is not known if a statistical difference exists between radiomic features from different tumor sites and how they affect machine learning model performance in endpoint prediction. To answer these questions, we extracted radiomic features from contrast-enhanced neck computed tomography scans (CTs) of 605 patients with HNSCC originating from the oral cavity, oropharynx, and hypopharynx/larynx. The difference in radiomic features of tumors from these sites was assessed using statistical analyses and Random Forest classifiers on the radiomic features with 10-fold cross-validation to predict tumor sites, nodal metastasis, and HPV status. We found statistically significant differences (*p*-value ≤ 0.05) between the radiomic features of HNSCC depending on tumor location. We also observed that differences in quantitative features among HNSCC from different locations impact the performance of machine learning models. This suggests that radiomic features may reveal biologic heterogeneity complementary to current gold standard histopathologic evaluation. We recommend considering tumor site in radiomic studies of HNSCC.

## 1. Introduction

Head and neck squamous cell carcinoma (HNSCC) is a heterogeneous malignancy constituting more than 95% of head and neck cancers and is the eighth leading cause of cancer death [1,2,3]. HNSCC is by far the most common mucosal malignancy of the head and neck arising most commonly from the oral cavity, oropharynx, larynx, and hypopharynx, and less commonly from the paranasal sinuses or the nasopharynx, although the latter is more commonly encountered in Asia.

Currently, clinical management of HNSCC is based primarily on TNM staging, which is essential for treatment planning and prognostication. The T-category is mostly based on primary tumor size and local disease extension, including invasion of critical organs; the N-category is determined by involvement of cervical lymph nodes, their size and laterality; the M-category dictates the presence of distant metastasis [4]. Although HNSCC TNM is largely anatomic based, in the most recent updated TNM staging in the 8th edition of the American Joint Committee on Cancer (AJCC) staging manual, HPV (human papilloma virus) status is incorporated as a molecular marker in the staging system of oropharyngeal tumors, with increased recognition of its important role in carcinogenesis and prognostication [5,6]. Epstein–Barr virus (EBV) drives pathogenesis of nasopharyngeal carcinoma by promoting cell growth, anti-apoptosis, and metastatic features and also is pathologically and clinically distinct from the more common mucosal HNSCCs [7]. Otherwise, similar histopathologic criteria and grading are used for oral cavity, oropharyngeal, hypopharyngeal, and laryngeal tumors. Oral cavity SCC is not as sensitive to radiotherapy and chemotherapy as oropharyngeal or laryngeal SCC; therefore, surgery is considered the primary treatment [4]. Laryngeal and hypopharyngeal cancers may be treated by chemoradiation or surgically depending on the tumor stage and based on the tumor board deliberations [4].

In addition to the traditional role of imaging for staging and post-treatment follow-up of HNSCC, there is increasing interest in the use of quantitative features extracted from the images or radiomic features for the characterization of HNSCC [8]. Radiomic features have demonstrated varying predictive values in recent years for molecular subgroup, HPV status, stage, locoregional control (LRC), progression-free survival (PFS), and overall survival (OS) of HNSCC [3,9,10,11,12,13,14,15,16,17,18,19]. Using machine learning algorithms, the quantitative image-based features extracted from cervical lymph nodes have also demonstrated the potential to predict the presence of metastasis in the lymph nodes [20,21].

Radiomic features can be influenced by the methodologies used at every stage of the analysis from image acquisition to feature extraction. Image quality and the extent of image preprocessing as well as the parameters used during image preprocessing can have a substantial impact on the reliability of radiomic features and the potential of these features capturing tumor heterogeneity [22,23]. These variabilities can lead to a lack of reproducibility and generalizability of the reported radiomic features [24,25]. We also hypothesize that the variation between radiomic features can also be influenced by the tumor site.

So far, most of the radiomic studies evaluating HNSCC are either heavily or exclusively based on analysis of tumors arising in the oropharynx or they combine tumors from different anatomical locations, with a few exceptions [10,11,17,19]. Given that the HNSCC genetic landscape, molecular pathogenesis, risk factors, and treatment response vary significantly across different primary tumors, we hypothesize that the quantitative radiomic features of HNSCC are site-dependent, and they might affect the performance of machine learning models in endpoint prediction. We study this hypothesis using two endpoints: nodal metastasis and HPV status. If this hypothesis is validated, site-specific radiomic features have the potential to reveal biologic heterogeneity that can be complementary to the current gold standard histopathologic classification of HNSCC.

## 2. Materials and Methods

### 2.1. Patients & Inclusion Criteria

This retrospective study was approved by the institutional ethical review boards, and the requirement of informed consent was waived. We retrospectively identified 605 consecutive patients diagnosed with primary pathology proven HNSCC with pretreatment computed tomography (CT) scans between 2010 and 2016, including 164 arising from oral cavity (OC), 200 oropharynx (OP), and 241 from larynx or hypopharynx (LHP). The tumor category (T-category) was determined by a multidisciplinary tumor board. The gold standard for lymph node involvement is pathological confirmation on resected lymph nodes, which was available for 186 patients; for the remaining cases without cervical lymph node dissection, the final decision of nodal category (N-category) at tumor board was used as the reference standard. The gold standard for HPV status is based on surrogate marker p16 immunohistochemistry (IHC) on biopsy or surgical samples. A summary of patients’ clinical information, lymph node metastasis, and HPV status is provided in Table 1.

### 2.2. Image Acquisition

CT scans of the neck were obtained after intravenous iodine contrast injection using multidetector scanners (Toshiba Aquilion 64, Aquilion one, Aquilion prime, Toshiba (Canon) Medical System, Tochigi-ken, Japan). Images were acquired from the frontal sinuses to the aortic arch, after double bolus intravenous injection of iopromide contrast (Ultravist, Bayer Healthcare, Whippany, NJ, USA). The first bolus of 50 mL was injected at a rate of 2 mL/s. After 120 s, a second bolus of 40 mL at 2 mL/s was injected. Images were acquired 10 s after regions of interest (ROI) at the aortic arch reached 160 HU. Images were acquired with a field of view of 220 mm in helical mode with a kVp of 120 and reconstructed axial images of 2 mm thickness using standard neck (soft tissue) kernel (FC43). A typical CT dose length product (DLP) between 500 and 700 mGy*cm was used.

**Figure 1:**
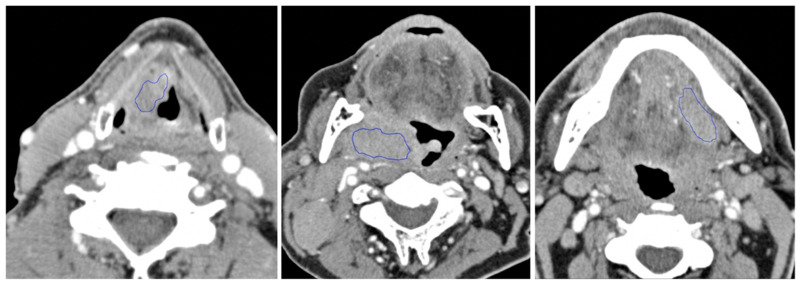
Examples of tumor segmentation using manually placed ROI contours in three different primary tumor locations: larynx/hypopharynx (left), oropharynx (middle), and oral cavity (right).

2D ROIs were manually drawn to segment the tumors on the axial contrast-enhanced neck CT images using TexRAD software (TexRAD; University of Sussex, Falmer, England), by a senior radiology resident, subsequently reviewed (with modifications if necessary) through consensus by two expert head and neck radiologists. Since the boundary between a tumor and nearby normal soft tissues is not always well defined, a conservative approach to contouring was taken to remain within the tumor, even at the risk of not including a small part of tumor edges. The slice with the largest area of the tumor was used for radiomic analysis, similar to multiple prior studies. Examples of tumor ROI contours are shown in Figure 1 for each of the anatomical locations (LHP, OP, and OC). Radiomic or “texture” features were extracted from the ROI using TexRAD. Six quantitative features were obtained based on gray-level intensity histogram, including the mean, standard deviation, mean of positive pixels, entropy, skewness, and kurtosis, for each of six spatial scale filters (SSF 0, 2, 3, 4, 5, 6) to highlight features ranging from fine to coarse textures [26], for a total of 36 quantitative features per ROI. Spatial scale filters were derived from a Laplacian of a Gaussian spatial band-pass filter to produce a series of derived images highlighting features at different anatomic spatial scales ranging from fine to coarse textures in the defined ROI. The SSF parameters of 2, 3, 4, 5, and 6 were used to enhance and extract fine to coarse imaging signal intensity features. An SSF value of 2 resulted in a fine texture feature with a radius of 2 mm, and an SSF value of 6 resulted in a coarse texture feature with a radius of 6 mm [26].

### 2.3. Statistical Analyses

To study the potential significance of the tumor site-specific information in predictive modeling, we assessed the association of tumor site with lymph node metastasis and HPV status. This was done by performing chi-square tests of independence to assess if an association exists between the anatomical site and the outcome of interest. The Python library Scipy v1.5.2 was utilized to perform these statistical tests.

For comparison of the radiomic features of tumors at different sites, the tumors were categorized into three groups based on the anatomical locations (OC, OP, and LHP). The 36 radiomic features extracted from each tumor from the three anatomical locations were then analyzed by t-Distributed Stochastic Neighbor Embedding (t-SNE) using the *MC3* v1.10.0 R package to characterize the clustering of these features according to tumor site as a 2-dimensional visualization. Multivariate analysis of variance (MANOVA) was done to determine if there was a statistically significant difference between the tumor site based on the selected quantitative features across spatial scale filters. A post hoc analysis of variance (ANOVA) was then performed to see which radiomic features differ across the three tumor sites (OC, OP, and LHP). An adjustment for multiple comparisons using Bonferroni correction [27] was performed to obtain an adjusted *p*-value, and an adjusted *p*-value less than 0.05 was considered statistically significant. We used R v4.0.2 along with the R package *rstatix* v0.6.0 and the base package *stats* for carrying out the statistical analysis for MANOVA and ANOVA.

### 2.4. Predictive Modeling of Different Outcomes Using Machine Learning

The 36 image-based radiomic features were used as input for building Random Forest (RF) [28] classifiers to develop prediction models for different outcomes. We hypothesize that tumor site-specific information can be reflected by radiomic features and consequently can affect the performance of machine learning models.

To assess if radiomic features contain site-specific information, we built an RF model to predict the anatomical site the tumors originated from using the radiomic features. This was done to determine if the site of the tumor can be accurately predicted using radiomic features alone.

**Figure 2:**
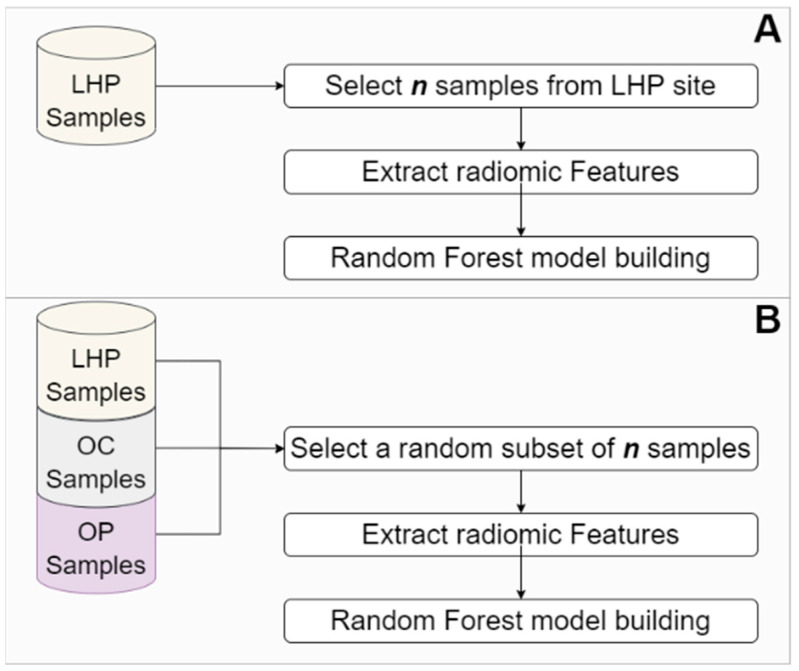
Panel (**A**) illustrates the model building process using samples from a site. Although panel A shows this for the LHP (larynx or hypopharynx) site, this process is also applicable to OC (oral cavity) or OP (oropharynx). Panel (**B**) illustrates the model building process using samples combined from all sites.

Next, to assess if this site-specific information can affect the performance of a machine learning model for endpoint prediction, we built RF models using samples of tumors from specific anatomical sites (LHP, OP, and OC) as well as RF models built using a site combined approach—i.e., no stratification based on tumor site. To do this, we developed RF classifiers for predicting nodal metastasis and HPV status in two different scenarios: (scenario 1) when data from a single tumor site were used for model development (Figure 2A) and (scenario 2) when data from all tumor sites were used for model development (Figure 2B). To prevent sample size from being the factor impacting model performance, datasets with an equal number of samples were used for both scenarios. Therefore, if site-specific information has no effect on model performance, one would expect that a model built using samples from all tumor sites at most leads to equal performance compared to models developed using samples from a single site. Each model was built, and its performance was measured based on accuracy, precision, recall, F1, and AUC score. To achieve statistically reliable results, we built 100 separate models, each with a dataset extracted based on scenario 2. For each site, the value of *n* selected for the number of samples used for building the site-combined models matched the number of available samples for that site. To alleviate the effect of the stochastic nature of model training on the results of the statistical analysis, we built 100 models for each sample site (scenario 1). Finally, we used a Wilcoxon rank-sum test to assess if there was any significant difference between the performance of the models built based on scenario 2 and the performance of the models developed based on scenario 1.

In order to build RF models, we followed the common practice for developing machine learning models [29]. To achieve an unbiased estimate of generalization error, 30% of the patients were randomly selected and set aside as the test group. Data from the remaining 70% were used for model development. The data partitioning in this paper was conducted in a stratified manner to preserve the distribution of samples for each endpoint of interest across training, validation, and test sets. Considering the relatively small number of features in comparison to sample size and the implicit mechanism of RF for selecting informative features, we did not use any explicit feature selection/reduction. Further, to overcome class imbalance, we used SMOTE (synthetic minority over-sampling technique) [30] on the training/validation set (but not on the test set).

We used grid search with a 10-fold cross-validation to train RF models and fine-tune their hyperparameters (see Appendix B). The hyperparameters that resulted in the best average F1-score on validation folds and the model trained using those hyperparameters were then selected. An unbiased evaluation of the model generalization error was then achieved using the test set, which had not been seen by the model. The performance measures reported in the paper were calculated based on the test set. Further, as the results on small classes in datasets with high class imbalance are not statistically reliable, we did not build stratified models for a site and endpoint of interest if the ratio between classes was equal to or smaller than 5:1. All machine learning models were trained and evaluated using Python v3.6.10 and *scikit-learn* v0.23.2.

## 3. Results

### 3.1. Association between Tumor Site and Lymph Node Metastasis and HPV Status

A chi-square test of independence was conducted to assess if an association exists between tumor site and nodal metastasis status. The resulting chi-square test suggested a significant relationship between tumor site and the nodal metastasis status, $\chi^{2}\left(2,\;N=604\right)=135.79$, p<0.001. Table 2 shows the conditional probability of nodal metastatic status given the tumor site of samples in our dataset. Only based on tumor site, for the OP samples, we can predict a nodal metastatic status with 87.5% accuracy; similarly, for LHP samples, we can make a nonmetastatic status prediction with a 67.6% accuracy.

It is well established that there is an association between OP primary site and HPV positive HNSCC clinically, and this was also reflected in our dataset. A chi-square test of independence was conducted to assess if there was an association between the tumor site and HPV status. The result of the chi-square test suggested a significant relationship between tumor site and the HPV status, $\chi^{2}\left(2,\;N=272\right)=63.01$, p<0.001. Table 3 shows the conditional probability of HPV status given the tumor site. Only using the knowledge of tumor site, for LHP samples in the dataset, we can predict HPV negative with 83.3% accuracy, i.e., if one predicts all LHP samples as HPV negative, such a prediction has 83.3% accuracy. Please note that there is no OC sample in the dataset with a positive HPV status.

### 3.2. Site-Specific Variation and Differences in Radiomic Features between Different Tumor Anatomic Sites

**Figure 3:**
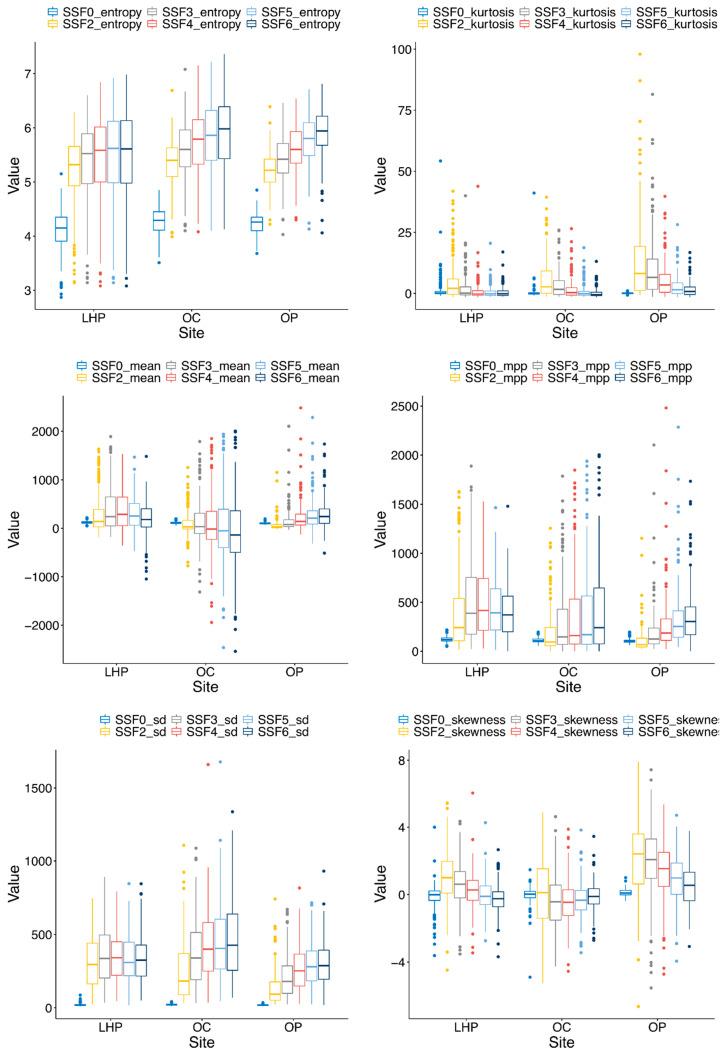
Box plots illustrating the distributions of quantitative features based on a gray-level intensity histogram including the mean, standard deviation, mean of positive pixels, entropy, skewness, and kurtosis. Each box plot represents the distributions of one feature for a different spatial scale filter (SSF 0, 2, 3, 4, 5, 6). LHP: larynx or hypopharynx; OC: oral cavity); OP: oropharynx.

**Figure 4:**
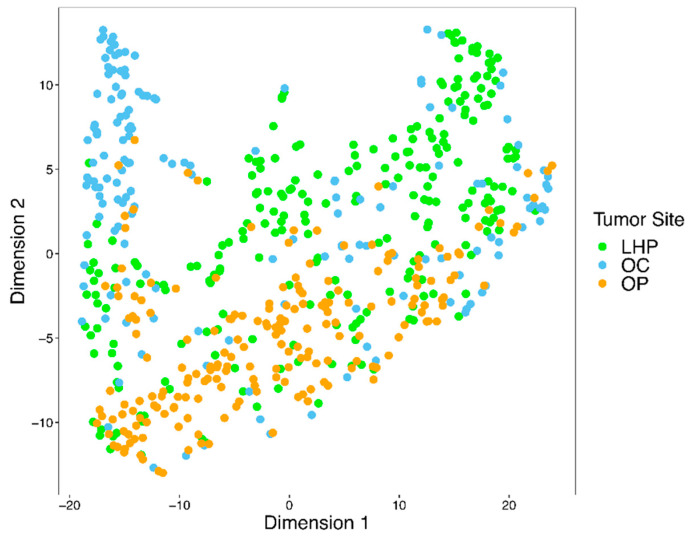
Clustering of texture features from three different anatomical locations (LHP: larynx or hypopharynx; OC: oral cavity); OP: oropharynx) using t-SNE with a perplexity value of 30.

Figure 3 illustrates the distributions of quantitative features used in this study. There were significant differences in radiomic features of HNSCC tumors from different anatomical sites based on MANOVA (*p*-value < 0.001) with partially separate clustering of tumors from OC, OP, and LHP sites on t-SNE (Figure 4). The post hoc results of ANOVA tests in Table A1 of Appendix A showed that radiomic features calculated based on average and standard deviation of intensity values, as well as radiomic features calculated based on entropy, skewness, and kurtosis of intensity values, were statistically different across tumor sites (adjusted *p*-value ≤ 0.05). As such, many of the features showed significant differences between tumor sites.

### 3.3. Radiomic Features Can Capture Tumor Site-Specific Information

As demonstrated above, radiomic features vary significantly across different tumor sites in HNSCC. In this section, we assess the hypothesis that these differences are substantial in a way that they can affect the performance of machine learning models. In the absence of site-specific information, one expects that a classification model developed for predicting three classes (LHP, OP, and OC), after addressing data imbalance, achieves an accuracy of about 0.333. Deviation from this value can be attributed to the tumor site-specific information encoded in radiomic features. To assess this hypothesis, we developed an RF model to classify samples based on their tumor sites. The model achieved an accuracy of 0.709, a precision of 0.705, a recall of 0.703, an F1 score of 0.703, and AUC of 0.869. The results show a large deviation from 0.333, which is the baseline performance assuming no association between radiomic features and tumor sites.

### 3.4. Stratification According to Primary Tumor Site Affects Prediction Performance of Lymph Node Metastasis and HPV Status

For each tumor site, the results of Wilcoxon rank-sum tests for comparing the performance of models built using samples from all sites combined and the model developed using samples from one single site are represented in Table 4. These include the results for RF models developed for lymph node metastasis prediction (LHP and OC) and the results for HPV status prediction for OP. Since the number of HPV samples for LHP and OC were not sufficient for developing RF models, we did not develop models for LHP and OC for HPV status prediction. As can be observed from Table 4, the calculated performance of models developed using samples from all tumor sites was significantly higher than that of models built only using samples from a single tumor site. This over-optimistic performance reflects an association between collected samples and the primary tumor sites rather than an association with the true tumor characteristics, as further elaborated on in the Discussion section. The accuracy, precision, recall, F1 score, and AUC score achieved for each model are available in the Supplementary File S1.

## 4. Discussion

We demonstrated that significant differences between quantitative features exist in HNSCC from different primary tumor anatomical locations, despite similar histopathologic classification as HNSCC, and that this difference has an impact on the performance of machine learning algorithms in predicting different endpoints of interest.

One may be inclined to assume that HNSCCs at different anatomic sites would have similar radiomic features based on their similar pathologic classification and grading. This has been used as justification for combining HNSCC tumors from different anatomic sites in many radiomic studies. However, our results suggest that this is not the case. Rather, the quantitative radiomic features of HNSCC are site-dependent and appear to reveal biologic heterogeneity. This challenges the common assumption and, importantly, raises concern for potential biases that, if not taken into account, will reduce the reliability and generalizability of radiomic-machine learning models.

These results of comparing the association between tumor site and lymph node metastasis and HPV status (Section 3.1) indicate that tumor site information significantly contributed to the performance of predictive models in our dataset. Although this site-specific information can boost the performance of predictive models, it might not always be medically informative and relevant to the phenotype of interest and might be just a direct or indirect consequence of the experimental design or data collection strategy. Further, the result of the classification model developed for predicting tumor sites (Section 3.3) showed a large deviation from the baseline performance, assuming no association between radiomic features and tumor sites. This large deviation indicates the existence of tumor site-specific information in radiomic features.

We therefore showed the importance of stratifying HNSCC tumors according to the tumor site when evaluating machine learning models developed using radiomic features. This is essential when developing machine learning models based on datasets with a class imbalance based on tumor sites, where the class imbalance resulted from a specific experimental design or data collection strategy and was not medically relevant to the endpoint under study.

We also demonstrated the feasibility of predicting nodal disease involvement using radiomic features from primary tumors, which has the potential to overcome some of the current challenge in imaging diagnosis of nodal metastasis and has an impact on future treatment planning. We expect that the findings in this research will help to develop more accurate and more generalizable machine learning models for various endpoints of interest involving diagnosis and treatment of HNSCC patients.

It should be noted that our results do not suggest, that for any given dataset, radiomic features from different tumor sites vary and affect the results of machine learning models. Rather, our experiment proves that such a scenario is possible, and therefore, radiomic studies should consider tumor sites as a potential confounding factor. We also acknowledge studies that have considered tumor site as a potential confounder in data analysis. For example, Huang et al. [3] used radiomic features extracted from pretreatment CT scans of 113 HNSCC patients to distinguish several molecular phenotypes. They reported that the tumor site has no effect on the extracted radiomic features and the resulting models. As another example, Feliciani et al. [31] studied the utility of radiomics features extracted from pretreatment ^18^F-FDG PET images to predict treatment outcome in 90 HNSCC patients treated with concurrent chemoradiation therapy. They reported that their model is tumor-site agnostic. Unfortunately, consideration of tumor site as a potential confounding factor is rare in radiomics studies. This consideration is another step toward developing generalizable radiomics models that can be utilized in clinical settings.

In this paper, we considered laryngeal and hypopharyngeal cancers under one group. The choice to consider laryngeal and hypopharyngeal cancers as one group was made due to the lower prevalence of these cancers and to avoid a highly imbalanced dataset. This is a limitation of our study; however, this does not affect the conclusions made in the paper. We suggest considering laryngeal and hypopharyngeal cancers as two different sites for predictive modeling applications.

In line with the published literature, our results support the utility of radiomic features for predicting different tumor characteristics, including the HPV status. It is crucial to note that for the prediction of HPV status, the class distribution of our dataset was balanced when combining tumors from all sites (HPV positive to negative ratio of 145:127). However, sample sets from individual sites were highly class-imbalanced (HPV positive to negative ratio of 11:55 for LHP, 0:9 for OC, and 134:63 for OP)—a reflection of the known association of HPV with the OP anatomical site—and the total number of cases with HPV status available is relatively small for LHP and OC. Considering the highly imbalanced nature of HPV status across tumor sites, if tumor site is ignored for analysis or evaluation, a machine learning model might predict all tumors from a specific site as HPV positive (or negative) just based on tumor site-specific features, rather than utilizing HPV-specific features for prediction.

Another example of the bias and potentially misleading performance can be seen in the results shown in Table 4, where the calculated performance of models developed using samples from all tumor sites was significantly higher than that of models built only using samples from a single tumor site, despite using the same number of samples for model building and having higher heterogeneity. This over-optimistic performance is due to an association between collected samples and the primary tumor sites rather than an association with the true tumor characteristics. The model built using only samples from one tumor site, however, can only rely on the radiomic feature predictive of the condition under study. These results highlight the importance of considering tumor site for constructing and evaluating machine learning models in head and neck cancer.

Our study has a number of limitations. First, the quantitative features we used comprised only first-order histogram-based texture features. We showed that these features were significantly different among different primary tumor locations and could affect the performance of machine learning models, but we should be careful to extrapolate this conclusion to all radiomic features used in current investigations. While we expect similar findings, for future studies, we suggest studying other radiomic features, including features based on Gray Level Cooccurrence Matrix, Gray Level Run Length Matrix, and Gray Level Size Zone Matrix [30]. These quantitative features should be tested for site-specific differences in HNSCC. Second, our study used 2D manual tumor segmentation instead of a 3D volumetric approach. This may not address intratumoral heterogeneity; however, no consensus in literature has been reached regarding the most optimal approach in this regard [32]. Third, class imbalance exists in individual tumor sites. This is unavoidable and mostly due to the nature of the tumor itself.

More specifically, we did not have a large enough sample size to test for LHP and OC individually for the prediction of HPV status. Future multicentered collaboration with larger and different datasets can potentially address this issue and provide external independent validation. It should, however, be noted that our study consists of a large cohort as compared to most published HNSCC studies.

## 5. Conclusions

In this research, we sought to assess if systematic differences exist between radiomic features resulting from different tumor sites in HNSCC and how these differences might affect machine learning model performance in endpoint prediction. Our statistical analysis showed that radiomic features are significantly different depending on the tumor location. This implies that radiomic features can capture site-specific information that could be a potential layer of information used by predictive models in the classification of HNSCC tumors, impacting machine learning model performance.

These findings suggest that tumor site should be considered when developing and evaluating radiomic-based predictive models in HNSCC. Our research is a step toward developing reliable and generalizable radiomic-machine learning models with the potential to be deployed in clinical settings.

## Figures and Tables

**Table 1:** Patient demographics and class distribution of lymph node metastasis (LN) and human papilloma virus (HPV) status across tumor sites—oral cavity (OC), oropharynx (OP), and larynx or hypopharynx (LHP). Note that for LN and HPV status, there are missing values for some patients.

	OC	OP	LHP	Total
Number of cases	164	200	241	605
Age	64 (24–90)	61 (33–87)	65 (27–88)	64 (24–90)
Sex (Male:Female)	111:53	161:39	201:40	473:132
LN (+/−)	93:70	175:25	78:163	346:258
HPV (+/−)	0:9	134:63	11:55	145:127

**Table 2:** Conditional probabilities of lymph node metastasis given the tumor site (LHP: larynx or hypopharynx; OC: oral cavity); OP: oropharynx).

Sites	Nonmetastatic	Metastatic
LHP	0.676	0.324
OC	0.429	0.571
OP	0.125	0.875

**Table 3:** Conditional probability of HPV (human papilloma virus) status given the tumor site (LHP: larynx or hypopharynx; OC: oral cavity); OP: oropharynx).

Sites	Negative	Positive
LHP	0.833	0.167
OC	1.00	0.00
OP	0.320	0.680

**Table 4:** The results of Wilcoxon rank-sum tests for comparing the performance of RF (Random Forest) models when all tumor sites were combined and used for model development (scenario 2) versus when only samples from one tumor site were used for model development (scenario 1). LN-LHP: lymph node metastasis prediction using features from larynx or hypopharynx sites; LN-OC: lymph node metastasis prediction using features from oropharynx site; HPV-OP: human papilloma virus status prediction using features from oropharynx site).

Endpoint-Site	Statistics	Accuracy	Precision	Recall	F1	AUC
**LN-LHP** (*N* = 241)	statistic	8.34	12.22	10.17	12.09	9.71
	*p*-value	<0.001	<0.001	<0.001	<0.001	<0.001
**LN-OC** (*N* = 163)	statistic	11.54	9.96	7.13	8.40	11.97
	*p*-value	<0.001	<0.001	<0.001	<0.001	<0.001
**HPV-OP** (*N* = 197)	statistic	11.12	0.32	7.73	4.06	11.67
	*p*-value	<0.001	<0.001	<0.001	<0.001	<0.001

## Data Availability

The source data presented in this study are not publicly available due to ethics and patient privacy related restrictions. The radiomic features extracted can be made available for reasonable requests by contacting the corresponding author.

## Supplementary Materials

The following are available online at https://www.mdpi.com/article/10.3390/cancers13153723/s1. File S1: An excel file containing the results of all RF models is provided.

## Appendix A

**Table A1:** Adjusted *p*-values for post hoc analysis of variance (ANOVA) among HNSCCs from three different sites.

Feature	Adjusted *p*-Value
SSF0_entropy	1.36 × 10^−6^
SSF0_kurtosis	4.02 × 10^−2^
SSF0_mean	7.99 × 10^−11^
SSF0_mpp	2.92 × 10^−11^
SSF0_sd	1.06 × 10^−3^
SSF0_skewness	1.97 × 10^−4^
SSF2_entropy	2.52 × 10^−1^
SSF2_kurtosis	3.01 × 10^−14^
SSF2_mean	2.64 × 10^−14^
SSF2_mpp	2.55 × 10^−21^
SSF2_sd	2.04 × 10^−23^
SSF2_skewness	9.41 × 10^−23^
SSF3_entropy	5.65 × 10^−2^
SSF3_kurtosis	2.57 × 10^−23^
SSF3_mean	8.38 × 10^−12^
SSF3_mpp	5.76 × 10^−18^
SSF3_sd	3.65 × 10^−16^
SSF3_skewness	6.04 × 10^−33^
SSF4_entropy	1.97 × 10^−3^
SSF4_kurtosis	6.90 × 10^−19^
SSF4_mean	4.38 × 10^−9^
SSF4_mpp	5.65 × 10^−9^
SSF4_sd	3.10 × 10^−13^
SSF4_skewness	1.30 × 10^−29^
SSF5_entropy	3.28 × 10^−6^
SSF5_kurtosis	9.52 × 10^−13^
SSF5_mean	1.08 × 10^−08^
SSF5_mpp	9.34 × 10^−02^
SSF5_sd	1.07 × 10^−13^
SSF5_skewness	8.98 × 10^−23^
SSF6_entropy	1.01 × 10^−9^
SSF6_kurtosis	1.17 × 10^−6^
SSF6_mean	9.33 × 10^−10^
SSF6_mpp	1.00
SSF6_sd	4.04 × 10^−14^
SSF6_skewness	1.15 × 10^−12^

## Appendix B

In this paper, we used the *RandomForestClassifier* from the ensemble module of the *scikit-learn* package. We utilized this classifier using a *GridSearchCV* from the *model_selection* module with the following arguments:

*n_estimators*: 100

*bootstrap*: True

*max_depth*: [4, 5, 6, 7]

*max_features*: [4, 5, 6]

*min_samples_leaf*: [4, 5, 6]

*oob_score*: True

The rest of the arguments were kept as default values. For each model, the combination of parameters that led to the maximum macro F1 score was used to build the trained classifier.
